# Double-Forming Mechanism of TaO_x_-Based Resistive Memory Device and Its Synaptic Applications

**DOI:** 10.3390/ma16186184

**Published:** 2023-09-13

**Authors:** Dongyeol Ju, Sunghun Kim, Subaek Lee, Sungjun Kim

**Affiliations:** Division of Electronics and Electrical Engineering, Dongguk University, Seoul 04620, Republic of Korea; judongyeol0117@gmail.com (D.J.); superdan919@gmail.com (S.K.); leesb981228@gmail.com (S.L.)

**Keywords:** resistive switching, neuromorphic system, synaptic plasticity, spike-timing-dependent plasticity

## Abstract

The bipolar resistive switching properties of Pt/TaO_x_/InO_x_/ITO-resistive random-access memory devices under DC and pulse measurement conditions are explored in this work. Transmission electron microscopy and X-ray photoelectron spectroscopy were used to confirm the structure and chemical compositions of the devices. A unique two-step forming process referred to as the double-forming phenomenon and self-compliance characteristics are demonstrated under a DC sweep. A model based on oxygen vacancy migration is proposed to explain its conduction mechanism. Varying reset voltages and compliance currents were applied to evaluate multilevel cell characteristics. Furthermore, pulses were applied to the devices to demonstrate the neuromorphic system’s application via testing potentiation, depression, spike-timing-dependent plasticity, and spike-rate-dependent plasticity.

## 1. Introduction

Because processing and memory components are physically separated, the conventional von Neumann architecture employed in computers encounters processing issues. Furthermore, modern CMOS-based electronic devices reach their scaling constraints owing to Moore’s law [1,2,3]. To overcome these problems, bioinspired neuromorphic computing is attracting great attention because of its high efficiency, low power consumption, and parallel data-processing feature [4,5]. The main focus of a neuromorphic computing system is to emulate the human brain’s synapses, in which large amounts of information move from one neuron to another. Many solid-state devices have been researched to mimic this system, and emerging nonvolatile devices are applicable candidates, including ferroelectric random-access memory (RAM) [6,7,8], magnetic RAM [9,10], phase-change RAM [11,12], and resistant RAM (RRAM) [13,14,15,16,17,18]. Among these, RRAM devices have benefits such as simple fabrication, high switching speeds, outstanding scalability, and high endurance, making them one of the most promising choices [19,20,21,22]. Moreover, the simple two-terminal structure of RRAMs, comprising a switching layer sandwiched between the top and bottom electrodes, most closely emulates the structure of a biological synapse [23]. Furthermore, applying different biases with different polarities causes a phenomenon termed the electro-resistance effect, where the resistance condition changes between a low-resistance state (LRS) and a high-resistance state (HRS), and which information is stored at 0 s and 1 s, respectively [24,25]. Various transition metal oxides have been employed as resistive switching insulators, including HfO_2_ [26], TiO_2_ [27,28,29], TaO_x_ [30], Al_2_O_3_ [31,32], and ZnO [30]. Extensive research has been conducted on TaO_x_, revealing it as a promising candidate for the resistive switching layer [33]. In addition, previous studies have indicated that TaO_x_ exhibits superior memory characteristics owing to its high endurance (>10^10^) [34], fast switching speed (<1 ns) [35], and good scalability (<30 nm) [36].

The resistive switching phenomena, which is the basis of RRAM, occurs due to the change in resistance states under an applied bias. For instance, in a valence change memory (VCM) device, the generation and rupture of the conducting filament is a key function in switching resistance [26,27,28]. The applied bias separates oxygen ions (O^2−^) and oxygen vacancies (Vo^+^). Then, due to the migration of oxygen ions under the applied electric field, the generated oxygen vacancies create a conductive filament that connects the top and bottom electrodes. Through the filament, a large current flows; thus, the resistance state is switched from a high resistance state (HRS) to a low resistance state (LRS). On the other hand, when the opposite bias is applied, reoxidation occurs in the conductive filament, and the filament is ruptured. Consequently, the filament ruptures and the device returns to HRS. The RRAM device stores memory in these two states, HRS and LRS, which can be consequently reproduced by applying sufficient bias [30]. To increase storage density, research has indicated that by applying multilevel cell (MLC) characteristics, a high storage density could be achieved due to multiple stable states between HRS and LRS. Research has indicated that multilevel cell (MLC) characteristics are evident in resistive switching devices, and they are key features that result in high storage density. This functionality allows devices to save data in the HRS, LRS, and between these two states by simply altering the compliance current (CC) and reset voltage [28,29,37]. Furthermore, the modulation of the memristive device’s conductance is both controllable and incremental, emulating the biological synapse. Here, the strength of the connection between the presynaptic and postsynaptic neurons is incrementally increased or decreased through input spikes by maintaining a history-dependent synaptic weight update [38,39]. Additionally, various synaptic functions can be emulated using pulse responses to assess the application of RRAM as a neuromorphic computing device. These functions include the potentiation and depression of short- and long-term memory (STM and LTM, respectively), spike-rate-dependent plasticity, and spike-time-dependent plasticity (STDP). Controllable conductance and synaptic weight changes can be monitored using these methods [40,41,42], while complex tests (such as pattern recognition systems through handwritten Modified National Institute of Standards and Technology (MNIST) datasets) are often conducted to evaluate the use of memristors as artificial synapses [43,44]. 

In this work, we studied a Pt/TaO_x_/InO_x_/indium tin oxide (ITO) device to investigate its potential for mimicking biological synapses. Bipolar gradual and uniform-resistive change behaviors were achieved with the MLC characteristic. Additionally, during the RF-sputtered deposition of the TaO_x_ layer, the diffusion of oxygen toward the ITO bottom electrode occurred, creating an InO_x_ layer. Due to the additional layer, the device exhibited unique forming behavior (termed double forming [45]) and self-compliance. Uniform switching during cycles (>10^2^) and retention (>10^4^) was also examined with gradual changes in potentiation and depression. The result of potentiation and depression was inserted into PRS using MNIST handwritten figures. Finally, synaptic functions such as long-term potentiation (LTP), long-term depression (LTD), STDP, and SRDP were emulated.

## 2. Experimental Section

The Pt/TaO_x_/InO_x_/ITO RRAM device was prepared using the following procedure. The bottom electrode was a commercially available ITO with a 30 nm thickness on a glass substrate (ITO/glass). Isopropyl alcohol and acetone were used to clean the surface, after which radio frequency (RF) reactive sputtering with a power of 150 W was used to deposit a 5 nm TaO_x_ layer on an ITO/glass substrate. The Ta source target was sputtered at room temperature with Ar (20 sccm) and O_2_ (6 sccm) at a pressure of 5 mTorr. An oxygen-rich InOx layer of 3 nm thickness was produced by the reactive–sputtering process owing to oxygen migration from TaO_x_ to ITO. Subsequently, a Ti adhesion layer of 1.5 nm thickness was formed by an e-beam evaporator. Finally, the e-beam evaporator was used to deposit Pt with a thickness of 100 nm, a deposition rate of 3 Å/s, and a pressure of 3.7 Torr. The liftoff process was performed to create patterned RRAM cells with a diameter of 100 μm. The electrical properties of Pt/TaO_x_/InO_x_/ITO were investigated using a Keithley 4200-SCS semiconductor parameter analyzer in the DC mode and a 4225-PMU ultrafast current–voltage (I–V) pulse module in the pulse mode. Furthermore, bias was applied to the top electrode (Pt), while the bottom electrode (ITO) was grounded at room temperature. The device properties (including cross-section analysis and elemental profiles) were determined using field emission transmission electron microscopy (TEM, JEOL JEMF200,Tokyo, Japan)) and X-ray photoelectron spectroscopy (XPS).

## 3. Results and Discussion

**Figure 1:**
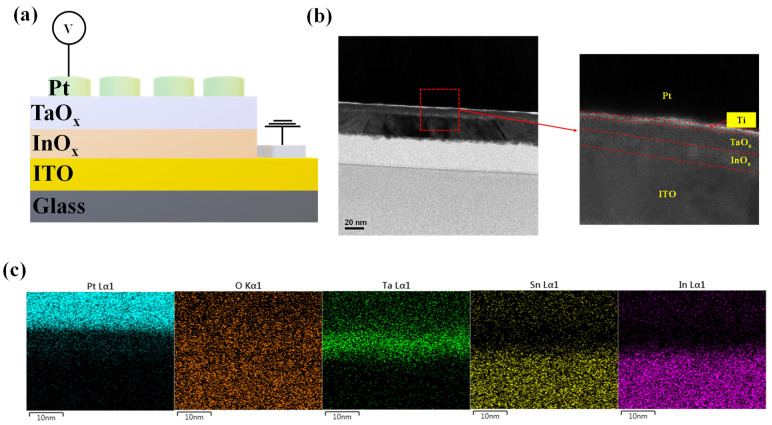
(**a**) Schematic illustration of the device’s structure. (**b**) Typical cross-sectional TEM image of the Pt/TaO_x_/InO_x_/ITO structure. (**c**) Component distribution: Pt, Ta, O, In, and Sn.

Figure 1a displays a schematic of the Pt/TaO_x_/InO_x_/ITO device. A TEM image was also used to verify the thickness of the device, as displayed in Figure 1b. Pt (100 nm thickness) and ITO (30 nm thickness) were verified using the TEM image. Furthermore, TaO_x_ and InO_x_ layers were observed between the Ti adhesion layer (1 nm thickness) and the ITO bottom electrode. Figure 1c depicts the elemental distribution of each layer, which was validated by an energy-dispersive X-ray spectroscopy line scan.

**Figure 2:**
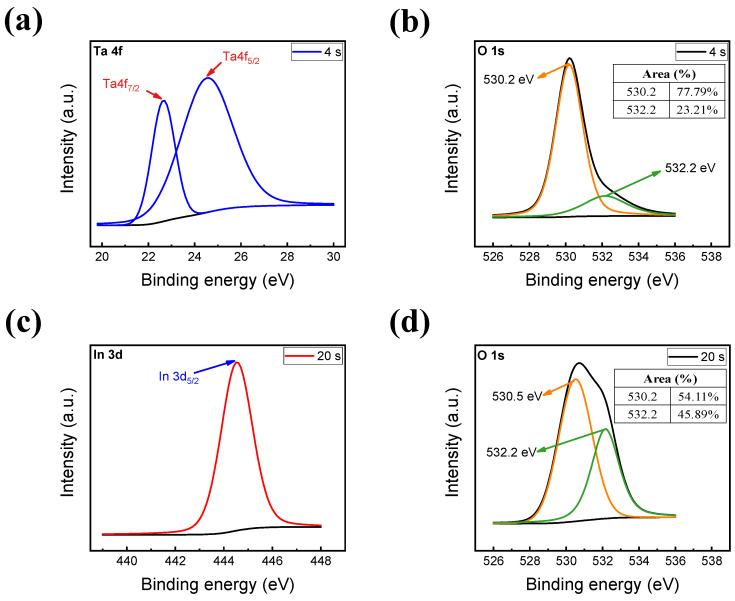
XPS spectra of (**a**) Ta 4f (**b**) and O 1s at an etch time of 4 s. (**c**) In 3d and (**d**) O 1s at an etch time of 20 s.

The chemical properties of the RRAM device are displayed in Figure 2. The insulating TaO_x_ and InO_x_ films were investigated in the XPS depth-profile mode. Figure 2a,b display the XPS spectra of Ta 4f and O 1s, respectively, for the TaO_x_ layer as the first insulator at 4 s. Two peaks of Ta4f_7/2_ and Ta4f_5/2_ were located around the binding energies of 22.52 and 25.13 eV, representing the Ta–O bonds. Additionally, the O 1s peak position of bulk TaO_x_ was located around 530.2 eV, indicating the existence of the TaO_x_ thin film. Furthermore, the second insulating layer (InO_x_) at an etch time of 20 s is depicted in Figure 2c,d. The spectral peaks of In3d_5/2_ and O 1s were located at approximately 444.81 and 530.5 eV, representing In–O bonding and the existence of an oxygen-distributed InO_x_ layer, respectively. In addition, the oxygen vacancy concentration of each insulating layer was inspected, as displayed in the insets of Figure 2b,d. The peak at 532.2 eV corresponds to the oxygen vacancies in the InO_x_ and TaO_x_ layers. Because of oxygen migration during the RF sputtering process, the percentage of oxygen vacancies in the TaO_x_ layer was 23.21% compared to 45.89% in the InO_x_ layer. Accordingly, fewer oxygen vacancies were stored in the TaO_x_ layer compared to the InO_x_ layer [45].

**Figure 3:**
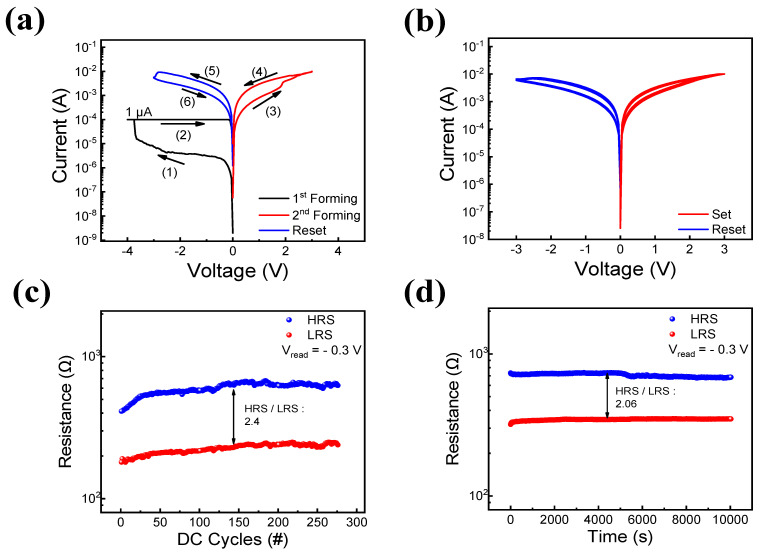
(**a**) Typical I–V curve. (**b**) Bipolar resistive switching ran 275 cycles. (**c**) Endurance test. (**d**) Retention test.

Figure 3 displays the electrical characteristics of Pt/TaO_x_/InO_x_/ITO under DC sweep conditions. In particular, Figure 3a displays the I–V curve, including the double-forming switching phenomenon, which is unlike the conventional RRAM operation. The “forming” process [46] (also known as dielectric soft breakdown) has been reported to occur once under applied bias, transforming the resistance state of the device from its initial state to LRS [47,48]. However, the RRAM device in this work required additional forming processes in the opposite bias to switch the resistance condition to LRS. The double-forming process can be divided into six steps: (1) the first forming process, (2) the medium state, (3) the second forming process, (4) LRS, (5) reset process, and (6) HRS, as displayed in Figure 3a [45]. By applying a voltage bias of −4 V and a CC of 100 μA to protect the device from hard breakdown, the first conducting filament was formed, and the device turned from its initial state into a medium state. Then, upon the application of a set voltage of 3 V and a reset voltage of −3 V, the device switched from the medium state to LRS and from LRS to HRS owing to the rupture and creation of a conduction path. The bipolar set and reset operations did not require CC, demonstrating self-compliance properties that could be implemented because of the ITO electrode [49,50]. Figure 3b depicts the successful resistive switching characteristic for 275 cycles. Under self-compliance conditions, a modest change in the current was detected, and the device maintained its original HRS and LRS without any significant degradation. As demonstrated in Figure 3a, there was an abrupt jump in the current at 2.1 V, similar to the first forming process at –3.8 V. However, a gradual change in current was exhibited in the set process shown in Figure 3b, and its window decreased from 4.55 to 2.21 at a read voltage of 0.3 V. Therefore, we applied the switching phenomena (3) and (4) as the second forming process with a forming voltage of 3 V. Figure 3c displays the endurance of the device with a read voltage of –0.3 V, where the device maintained its HRS and LRS. Additionally, we investigated the data retention capability of the device, as displayed in Figure 3d. These results indicate that the device maintained its HRS and LRS for 10^4^ s without degradation, demonstrating its good nonvolatile properties.

**Figure 4:**
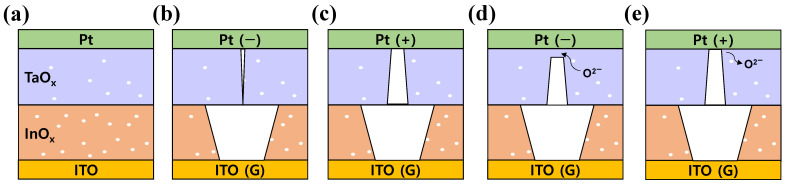
Schematic description of the conduction mechanism of the double-forming process of the Pt/TaO_x_/InO_x_/ITO RRAM device in the (**a**) Initial state, (**b**) First forming process, (**c**) Second forming process, (**d**) Reset process, and (**e**) Set process.

Figure 4 depicts the conduction mechanism of the Pt/TaO_x_/InO_x_/ITO device, where the white dot in the figure represents the implementation of lattice oxygen vacancy. Lin et al. reported that the physical size of the conductive filament could be determined by the existence of lattice oxygen vacancy, where the size of the conducting filament in the oxygen-vacancy-deficient layer was narrower than that in an oxygen-vacancy-rich layer [51]. Furthermore, Huang et al. explained the double-forming mechanism of bilayer-structured resistive switching devices using an asymmetric conductive filament [45]. Based on these previous studies, it can be implied that implementing different amounts of existing lattice oxygen vacancies in the TaO_x_ and InO_x_ layers constructs two different sizes of conducting filaments in each insulating layer. Through the connection of two asymmetric filaments, the device switches to LRS, observing the double-forming mechanism. A thick filament was formed in the oxygen-vacancy-rich InO_x_ layer, whereas a relatively narrow filament was formed in the oxygen-vacancy-deficient TaO_x_ layer. When negative voltage was applied to the top electrode, the first forming process occurred. Redox reactions then caused the separation of oxygen vacancies (V_o_^+^) and oxygen ions (O^2−^). These separated oxygen ions were then repelled away from the top electrode due to an applied bias. Thus, the generated defects (oxygen vacancies) accumulated and formed conductive filaments of different sizes in the InO_x_ and TaO_x_ layers, as depicted in Figure 4b. However, CC limited the thickening of the conduction path in the TaO_x_ layer, implying that the current decreased in step (2) of Figure 3a. Then, under a positive voltage applied to the top electrode, oxygen ions migrated toward the bottom electrode and were repelled back toward the top electrode due to the electric field. Due to the additional generation of oxygen vacancies in this process, a conductive filament in TaO_x_, with its thickest part formed at the interface of InO_x_ and TaO_x_ could be completed. Consequently, the asymmetric shape of the conductive filament (Figure 4c) could be constructed. In this second forming process, the device finally changed to LRS (as in step (4)), and repeatable resistive switching phenomena could be observed in the device. The reset process occurred when a negative voltage was applied to Pt. Oxygen ions drifted toward the TaO_x_ layer to recombine with the oxygen vacancies. The weakest part of the filament, at the interface of Pt/TaO_x_, was ruptured due to the recombination of the oxygen ion and vacancy, assisted with local joule heating [52,53]. The schematic of the reset process is shown in Figure 4d, where the rupture of the weakest part of the conductive filament could be observed. On the other hand, when a positive voltage was applied to the top electrode, oxygen ions migrated toward the top electrode due to the applied electric field, leaving the oxygen vacancies. Thus, defects again accumulated, and the reconstruction of conduction paths occurred. Thus, a large current flowed through the filament, altering the device state to LRS.

Further, we investigated the MLC characteristics of the device. For the application of the device as a synaptic device, MLC characteristics are important for implementing the multiple weights of each synapse in an artificial neural network [54,55]. Additionally, it features high storage density and introduces multiple data storage areas. Two types of voltage bias schemes are usually applied to investigate MLC characteristics: (i) controlling the reset voltage and (ii) controlling the CC.

**Figure 5:**
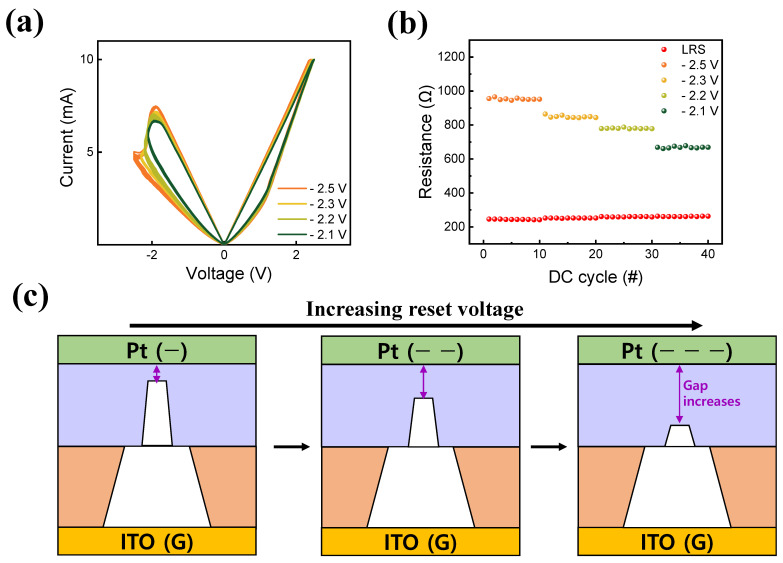
(**a**) MLC obtained by controlling the reset voltage and (**b**) DC endurance performance. (**c**) Filament schematics.

Figure 5 presents the method for controlling the reset voltage. In Figure 5a, the I–V curve of a Pt/TaO_x_/InO_x_/ITO device with four reset voltages is displayed, which reveals that adjusting the reset voltage results in multiple HRSs while maintaining the LRS as constant. This effect is caused by the varying rate of ruptured filaments under different reset voltages [56]. As the reset voltage increases, more oxygen ions are repelled from the top electrode, changing the degree of recombination rate. Therefore, the gap between the Pt top electrode and the conducting filament increases, resulting in decreased I_HRS_ [Figure 5c]. Figure 5b depicts the cycling endurance with varying reset voltages. As stated previously, increasing the reset voltages increased HRS, resulting in a lower I_HRS_, while LRS remained fairly constant.

**Figure 6:**
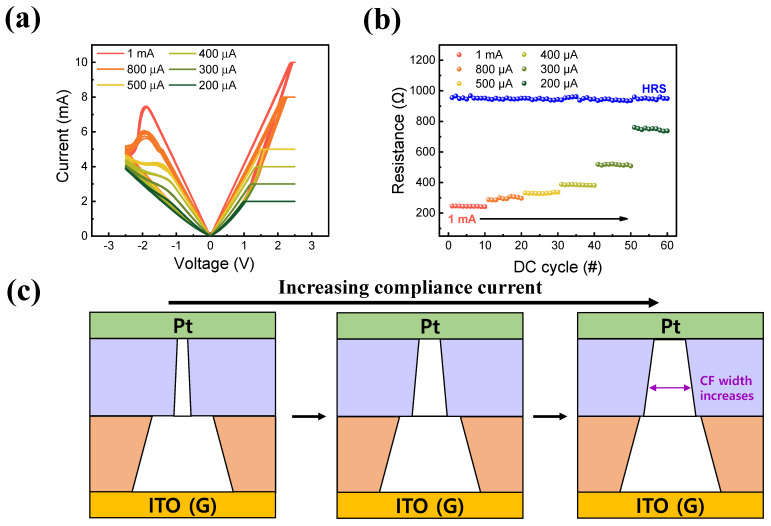
(**a**) MLC obtained by the controlling compliance current and (**b**) DC endurance performance. (**c**) Filament schematics.

Another method of obtaining MLC characteristics was controlling CC during the set operation. Figure 6a displays the I–V curve of a Pt/TaO_x_/InO_x_/ITO device with six CC settings. Here, CC was adjusted from 200 μA to 1 mA, and the reset voltage was maintained at −2.5 V. Evidently, increasing CC reduced LRS while maintaining a constant HR. This phenomenon was considered to be induced by an increase in CC, which caused an increase in the current flow in the LRS state. Figure 6c presents a filament schematic of the controlled CC. As the CC increased, the width of the conducting filament also increased. Then, more electrons could move through the enlarged conducting path, resulting in a larger I_LRS_ [44,57]. Consequently, I_LRS_ increased, while I_HRS_ remained unchanged. Figure 6b illustrates a 10-cycle endurance test while applying different CCs. Here, the resistance of the LRS decreased while CC increased.

**Figure 7:**
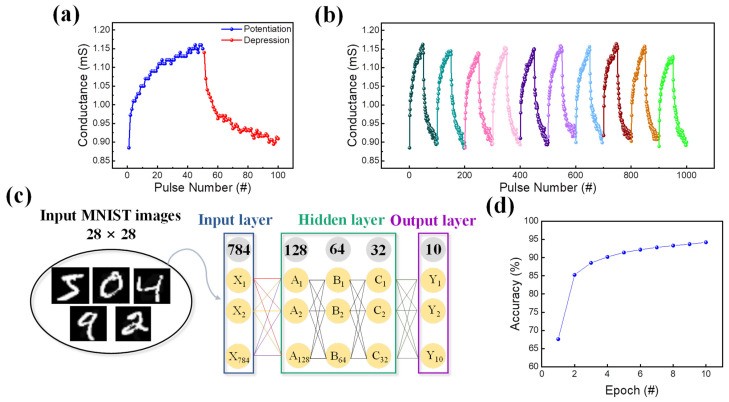
(**a**) Potentiation and depression. (**b**) Potentiation and depression run for 10 cycles. (**c**) Schematic illustration of a DNN for numerical number recognition containing the input (784 neurons), hidden (3 layers), and output (10 neurons) layers. (**d**) Simulated recognition accuracy using the MNIST numerical datasets of raining images, with approximately 94% recognition accuracy for the Pt/TaO_x_/InO_x_/ITO memristor.

Next, a scheme of 100 consecutive pulses was applied to evaluate the synaptic characteristics of Pt/TaO_x_/InO_x_/ITO. The pulse train comprised 50 identical potentiation and depression pulses each. For potentiation, the pulse width and amplitude were 100 μs and 2 V, respectively, compared to 50 μs and −2.7 V for depression. Figure 7a depicts the result of the applied pulse scheme, which demonstrated a linear rise and decay of conductance. Then, to test the synaptic reproducibility, LTP and LTD were explored using 10-cycle potentiation and depression pulse methods [58]. The results are depicted in Figure 7b, where identical conductance changes could be observed. In other words, the conductance levels after each pulse application favorably maintained their states under repetitive operation, proving the applicability of the device to mimic the human brain. Additionally, a pattern recognition test using handwritten digits from an MNIST dataset was conducted to check the further application of the device as a synaptic device. The training was conducted with unclear images, and the gradual and symmetric conductance changes in potentiation and depression resulted in clearer images with higher accuracy [59,60]. As shown in Figure 7c, the deep neural network (DNN) comprised 784 input neurons, 3 hidden layers, and 10 output neurons. Each of these three hidden layers had 128, 64, and 32 neurons, and the backpropagation method was employed to improve accuracy. To determine linearity and accuracy, the potentiation and depression depicted in Figure 7a were converted into an MNIST handwritten number of 28 × 28 pixels and applied to the neural network. The result of the number recognition is illustrated in Figure 7d, where the highest obtained accuracy was 94.21%.

**Figure 8:**
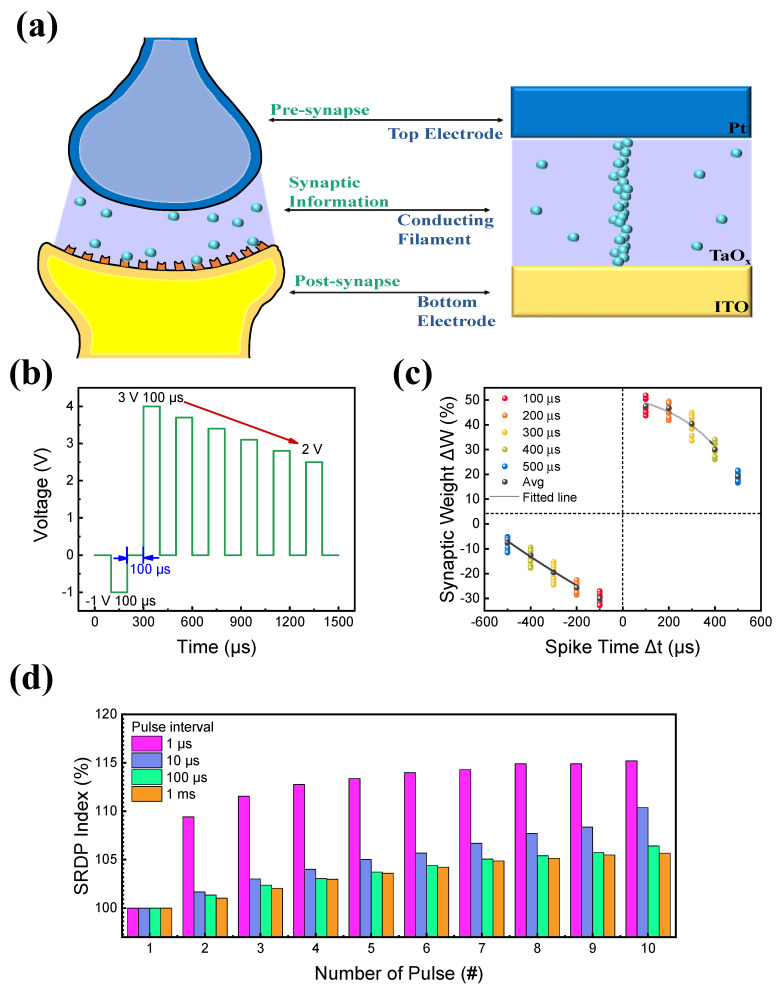
(**a**) Schematic illustration of the human synaptic neural structure. (**b**) Pulse schematic. (**c**) Result of the STDP measurement. (**d**) Result of the SRDP measurement.

Finally, the Hebbian learning rules of synapses and neurons were tested on a Pt/TaO_x_/InO_x_/ITO device to investigate its ability to mimic a biological synapse [61]. Figure 8a illustrates the RRAM device mimicking the human synapse. Here, the top and bottom electrodes mimic pre- and post-spikes, while synaptic information between the neurons varied due to the conducting filament connecting the top and bottom electrodes [62,63]. STDP is composed of two parts of synaptic variance: LTP and LTD. For example, LTP occurs when the pre-spike exceeds the post-spike, while LTD occurs when the post-spike exceeds the pre-spike. These synaptic investigations were conducted by applying the same pulse train to the pre-and post-spikes. A pulse train with a pulse width of 100 μs was applied to Pt/TaO_x_/InO_x_/ITO, as illustrated in Figure 8b. The pulse application was conducted with a difference in the time interval, which is termed spike timing difference Δt (Δt = t_pre_ − t_post_). During LTP (Δt > 0), a set of positive pulse trains was applied, which induced a decrease in the resistance of the devices. Further, negative pulse trains were applied during LTD (Δt < 0), inducing an increase in the resistance of the devices. The conductance was acquired because the pulse application was input into the formula below to convert it into synaptic weight (ΔW), which represented the spike connection:

$$\Delta W=\frac{G_{f}-G_{i}}{G_{i}}\times 100\left(\%\right),$$

where G_i_ and G_f_ represent the conductance of the initial value before and the final value after applying the pulse trains, respectively. Figure 8c presents the experimental data of STDP obtained from the device. For Δt > 0, the weight change increased continuously with decreasing time intervals, and LTP was obtained. By contrast, for Δt < 0, the weight change decreased, and LTD was obtained. This result proves the successful experimental demonstration of the STDP learning rule with synaptic weight changes at different spike times using the proposed Pt/TaO_x_/InO_x_/ITO memristor device, favorably mimicking a biological synapse [23]. Additionally, another Hebbian learning rule, SRDP, was tested to obtain the device’s frequency-dependent characteristics [64]. Ten consequent pulses were applied to the Pt/TaO_x_/InO_x_/ITO device with the same pulse width and amplitude of 100 μs and 2 V. These pulse intervals varied from 1 μs to 1 ms, as depicted in Figure 8d. The term SRDP Index is calculated as:

$$SRDPIndex=\frac{I_{n}}{I_{i}}\times 100\left(\%\right)$$

where I_n_ and I_i_ represent the current of the initial value before and the current after applying consequent pulse trains. The result indicates that when the pulse interval is small, the device response rapidly increases, successfully emulating SRDP behavior.

## 4. Conclusions

In this study, Pt/TaO_x_/InO_x_/ITO exhibited bipolar resistive switching characteristics under double-forming conditions. The device also exhibited acceptable endurance (>10^2^) and retention (>10^4^) properties, with multilevel characteristics, which were demonstrated by varying the reset voltage and CC. Furthermore, pulse trains were applied to reveal their neuromorphic system characteristics. The results of potentiation and depression, MNIST pattern recognition, STDP, and SRDP proved their potential for future applications as a neuromorphic device.

## Data Availability

Not applicable.

## References

[1] Baek M.-H., Kim H. (2023). Polysilicon-Channel Synaptic Transistors for Implementation of Short- and Long-Term Memory Characteristics. Biomimetics.

[2] Li H., Gao B., Chen Z., Zhao Y., Huang P., Ye H., Liu L., Liu X., Kang J. (2015). A learnable parallel processing architecture towards unity of memory and computing. Sci. Rep..

[3] Milo V., Malavena G., Compagnoni C.M., Ielmini D. (2020). Memristive and CMOS devices for neuromorphic computing. Materials.

[4] Woo J., Moon K., Song J., Lee S., Kwak M., Park J., Hwang H. (2016). Improved synaptic behavior under identical pulses using AlO_x_/HfO_2_ bilayer RRAM array for neuromorphic systems. IEEE Electron. Device Lett..

[5] Wu W., Wu H., Gao B., Deng N., Yu S., Qian H. (2017). Improving Analog Switching in HfOx-Based Resistive Memory with a Thermal Enhanced Layer. IEEE Electron. Device Lett..

[6] Hiroshi I. (2012). Ferroelectric Random Access Memories. J. Nanosci. Nanotechnol..

[7] Yoshihiro A., Ishiwara H. (2004). Current Status of Ferroelectric Random-Access memory. MRS Bull..

[8] Mikolajick T., Slesazeck S., Park M.H., Schroeder U. (2018). Ferroelectric hafnium oxide for ferroelectric random-access memories and ferroelectric field-effect transistors. MRS Bull..

[9] Krzysteczko P., Münchenberger J., Schäfers M., Reiss G., Thomas A. (2012). The memristive magnetic tunnel junction as a nanoscopic synapse-neuron system. Adv. Mater..

[10] Vincent A.F., Larroque J., Locatelli N., Romdhane N.B., Bichler O., Gamrat C., Zhao W.S., Klein J.O., Galdin-Retailleau S., Querlioz D. (2015). Spin-transfer torque magnetic memory as a stochastic memristive synapse for neuromorphic systems. IEEE Trans. Biomed. Circuits Syst..

[11] Kuzum D., Jeyasingh R.G.D., Lee B., Wong H.S.P. (2012). Nanoelectronic programmable synapses based on phase change materials for brain-inspired computing. Nano Lett..

[12] Burr G.W., Shelby R.M., Sidler S., Di Nolfo C., Jang J., Boybat I., Shenoy R.S., Narayanan P., Virwani K., Giacometti E.U. (2015). Experimental Demonstration and Tolerancing of a Large-Scale Neural Network (165,000 Synapses) Using Phase-Change Memory as the Synaptic Weight Element. IEEE Trans. Electron Devices.

[13] Gao B., Bi Y., Chen H.Y., Liu R., Huang P., Chen B., Liu L., Liu X., Yu S., Wong H.S.P. (2014). Ultra-low-energy three-dimensional oxide-based electronic synapses for implementation of robust high-accuracy neuromorphic computation systems. ACS Nano.

[14] Wang I.T., Chang C.C., Chiu L.W., Chou T., Hou T.H. (2016). 3D Ta/TaO_x_/TiO_2_/Ti synaptic array and linearity tuning of weight update for hardware neural network applications. Nanotechnology.

[15] Ambrogio S., Balatti S., Milo V., Carboni R., Wang Z.Q., Calderoni A., Ramaswamy N., Ielmini D. (2016). Neuromorphic Learning and Recognition with One-Transistor-One-Resistor Synapses and Bistable Metal Oxide RRAM. IEEE Trans. Electron Devices.

[16] Wang H., Yan X. (2019). Overview of Resistive Random Access Memory (RRAM): Materials, Filament Mechanisms, Performance Optimization, and Prospects. PhysStatus Solidi-Rapid Res. Lett..

[17] Li C., Hu M., Li Y., Jiang H., Ge N., Montgomery E., Zhang J., Song W., Dávila N., Graves C.E. (2018). Analogue signal and image processing with large memristor crossbars. Nat. Electron..

[18] Wang Z., Joshi S., Savel’Ev S., Song W., Midya R., Li Y., Rao M., Yan P., Asapu S., Zhuo Y. (2018). Fully memristive neural networks for pattern classification with unsupervised learning. Nat. Electron..

[19] Park J., Kwak M., Moon K., Woo J., Lee D., Hwang H. (2016). TiO_x_-Based RRAM Synapse with 64-Levels of Conductance and Symmetric Conductance Change by Adopting a Hybrid Pulse Scheme for Neuromorphic Computing. IEEE Electron Device Lett..

[20] Moon K., Kwak M., Park J., Lee D., Hwang H. (2017). Improved Conductance Linearity and Conductance Ratio of 1T2R Synapse Device for Neuromorphic Systems. IEEE Electron Device Lett..

[21] Mehonic A., Kenyon A.J. (2016). Emulating the electrical activity of the neuron using a silicon oxide RRAM cell. Front. Neurosci..

[22] Liu J., Yang H., Ma Z., Chen K., Zhang X., Huang X., Oda S. (2017). Characteristics of multilevel storage and switching dynamics in resistive switching cell of Al_2_O_3_/HfO_2_/Al_2_O_3_ sandwich structure. J. Phys. D Appl. Phys..

[23] Park J., Park H., Chung D., Kim S. (2022). Dynamic and Static Switching in ITO/SnO_x_/ITO and Its Synaptic Application. Int. J. Mol. Sci..

[24] Wei X., Huang H., Ye C., Wei W., Zhou H., Chen Y., Zhang R., Zhang L., Xia Q. (2019). Exploring the role of nitrogen incorporation in ZrO_2_ resistive switching film for enhancing the device performance. J. Alloys Compd..

[25] Liu H.C., Tang X.G., Liu Q.X., Jiang Y.P., Li W.H., Guo X.B., Tang Z.H. (2020). Bipolar resistive switching behavior and conduction mechanisms of composite nanostructured TiO_2_/ZrO_2_ thin film. Ceram. Int..

[26] Puglisi F.M., Pavan P., Padovani A., Larcher L. (2014). A study on HfO_2_ RRAM in HRS based on I-V and RTN analysis. Solid-State Electron..

[27] Kim K.M., Park T.H., Hwang C.S. (2015). Dual conical conducting filament model in resistance switching TIO_2_ thin films. Sci. Rep..

[28] Cho H., Kim S. (2020). Short-Term Memory Dynamics of TiN/Ti/TiO_2_/SiO_x_/Si Resistive Random Access Memory. Nanomaterials.

[29] Sedghi N., Li H., Brunell I.F., Dawson K., Potter R.J., Guo Y., Gibbon J.T., Dhanak V.R., Zhang W.D., Zhang J.F. (2017). The role of nitrogen doping in ALD Ta_2_O_5_ and its influence on multilevel cell switching in RRAM. Appl. Phys. Lett..

[30] Park M., Jeon B., Park J., Kim S. (2022). Memristors with Nociceptor Characteristics Using Threshold Switching of Pt/HfO_2_/TaO_x_/TaN Devices. Nanomaterials.

[31] Ryu H., Kim S. (2020). Synaptic Characteristics from Homogeneous Resistive Switching in Pt/Al_2_O_3_/TiN Stack. Nanomaterials.

[32] Asapu S., Maiti T. (2017). Multifilamentary conduction modeling in transition metal oxide-based rram. IEEE Trans. Electron Devices.

[33] Lee M.H., Kim K.M., Kim G.H., Seok J.Y., Song S.J., Yoon J.H., Hwang C.S. (2010). Study on the electrical conduction mechanism of bipolar resistive switching TiO_2_ thin films using impedance spectroscopy. Appl. Phys. Lett..

[34] Yang J.J., Zhang M.X., Strachan J.P., Miao F., Pickett M.D., Kelley R.D., Medeiros-Ribeiro G., Williams R.S. (2010). High switching endurance in TaO_x_ memristive devices. Appl. Phys. Lett..

[35] Torrezan A.C., Strachan J.P., Medeiros-Ribeiro G., Williams R.S. (2011). Sub-nanosecond switching of a tantalum oxide memristor. Nanotechnology.

[36] Lee M.J., Lee C.B., Lee D., Lee S.R., Chang M., Hur J.H., Kim Y.B., Kim C.J., Seo D.H., Seo S. (2011). A fast, high-endurance and scalable non-volatile memory device made from asymmetric Ta_2_O_5_-xx/TaO_2_-xbilayer structures. Nat. Mater..

[37] Deuermeier J., Kiazadeh A., Klein A., Martins R., Fortunato E. (2019). Mulit-level cell properties of a bilayer Cu_2_O/Al_2_O_3_ resistive switching device. Nanomaterials.

[38] Malenka R.C. (1994). Synaptic plasticity in the hippocampus: LTP and LTD. Cell.

[39] Malenka R.C., Francisco S. (1995). Review: LTP and LTD: Dynamic and interactive processes of synaptic plasticity. Neuroscientist.

[40] ASokolov S., Jeon Y.R., Ku B., Choi C. (2020). Ar ion plasma surface modification on the heterostructured TaO_x_/InGaZnO thin films for flexible memristor synapse. J. Alloys Compd..

[41] Kim M.K., Lee J.S. (2018). Short-Term Plasticity and Long-Term Potentiation in Artificial Biosynapses with Diffusive Dynamics. ACS Nano.

[42] Prezioso M., Bayat F.M., Hoskins B., Likharev K., Strukov D. (2016). Self-Adaptive Spike-Time-Dependent Plasticity of Metal-Oxide Memristors. Sci. Rep..

[43] Zhang Y., Li Y., Wang X., Friedman E.G. (2017). Synaptic Characteristics of Ag/AgInSbTe/Ta-Based Memristor for Pattern Recognition Applications. IEEE Trans. Electron Devices.

[44] Klimo M., Such O., Skvarek O., Fratrik M. (2014). Memristor-based pattern matching. Semicond. Sci. Technol..

[45] Huang C.Y., Huang C.Y., Tsai T.L., Lin C.A., Tseng T.Y. (2014). Switching mechanism of double forming process phenomenon in ZrO_x_/HfO_y_ bilayer resistive switching memory structure with large endurance. Appl. Phys. Lett..

[46] Degraeve R., Fantini A., Raghavan N., Goux L., Clima S., Govoreanu B., Belmonte A., Lineyen D., Jurczak M. (2015). Causes and consequences of the stochastic aspect of filamentary RRAM. Microelectron. Eng..

[47] Oh I., Pyo J., Kim S. (2022). Resistive Switching and Synaptic Characteristics in ZnO/TaON-Based RRAM for Neuromorphic System. Nanomaterials.

[48] Athena F.F., West M.P., Hah J., Hanus R., Graham S., Vogel E.M. (2022). Towards a better understanding of the forming and resistive switching behavior of Ti-doped HfO_x_ RRAM. J. Mater. Chem..

[49] Ye C., Zhan C., Tsai T.M., Chang K.C., Chen M.C., Chang T.C., Deng T., Wang H. (2014). Low-power bipolar resistive switching TiN/HfO_2_/ITO memory with self-compliance current phenomenon. Appl. Phys. Express.

[50] Lin C.Y., Chang K.C., Chang T.C., Tsai T.M., Pan C.H., Zhang R., Liu K.H., Chen H.M., Tseng Y.T., Hung Y.C. (2015). Effects of varied negative stop voltages on current self-compliance in indium tin oxide resistance random access memory. IEEE Electron. Device Lett..

[51] Lin M.H., Wu M.C., Huang C.Y., Lin C.H., Tseng T.Y. (2010). High-speed and localized resistive switching characteristics of double-layer SrZrO_3_ memory devices. J. Phys. D Appl. Phys..

[52] Zhao L., Chen H., Wu S., Jiang Z., Yu S., Hou T., Wong H.S., Nishi Y. Improved multi-level control of RRAM using pulse-train programming. Proceedings of the Technical Program—2014 International Symposium on VLSI Technology, Systems and Application (VLSI-TSA).

[53] Tsai T.L., Chang H.Y., Lou J.J.C., Tseng T.Y. (2016). A high performance transparent resistive switching memory made form ZrO_2_/AlON bilayer structure. Appl. Phys. Lett..

[54] Ling H., Yi M., Nagai M., Xie L., Wang L., Hu B., Huang W. (2017). Controllable Organic Resistive Switching Achieved by One-Step Integration of Cone-Shaped contact. Adv. Mater..

[55] Bai Y., Wu H., Wu R., Zhang Y., Deng N., Yu Z., Qian H. (2014). Study of multi-level characteristics for 3D vertical resistive switching memory. Sci. Rep..

[56] Milo V., Zambelli C., Olivo P., Pérez E., Mahadevaiah M.K., Ossorio O.G., Wenger C., Ielmini D. (2019). Multilevel HfO_2_-based RRAM devices for low-power neuromorphic networks. APL Mater..

[57] Prakash A., Hwang H. (2016). Multilevel cell storage and resistance variability in resistive random access memory. Phys. Sci. Rev..

[58] Wang Z., Yin M., Zhang T., Cai Y., Wang Y., Yang Y., Huang R. (2016). Engineering incremental resistive switching in TaO_x_ based memristors for brain-inspired computing. Nanoscale.

[59] Jena A.K., Sahu M.C., Mohanan K.U., Mallik S.K., Sahoo S., Pradhan G.K., Sahoo S. (2023). Bipolar Resistive Switching in TiO_2_ Artificial Synapse Mimicking Pavlov’s Associative Learning. ACS Appl. Mater. Interfaces.

[60] Ju D., Kim J., Kim S. (2023). Highly uniform resistive switching characteristics of Ti/TaO_x_/ITO memeristor devices for neuromorphic system. J. Alloys Compd..

[61] Caporale N., Dan Y. (2008). Spike timing–dependent plasticity: A Hebbian learning rule. Annu. Rev. Neurosci..

[62] Zhang S.R., Zhou L., Mao J.Y., Ren Y., Yang J.Q., Yang G.H., Zhu X., Han S.T., Roy V.A.L., Zhou Y. (2019). Artificial Synapse Emulated by Charge Trapping-Based Resistive Switching Device. Adv. Mater. Technol..

[63] Wang Z., Wu H., Burr G.W., Hwang C.S., Wang K.L., Xia Q., Yang J.J. (2020). Resistive switching materials for information processing. Nat. Rev. Mater..

[64] Huang F., Ke C., Li J., Chen L., Yin J., Li X., Wu Z., Zhang C., Wu F., Wu Y. (2023). Controllable Resistive Switching in ReS_2_/WS_2_ Heterostructure for Nonvolatile Memory and Synaptic Simulation. Adv. Sci..

